# Supplementary material for: Taxifolin Inhibits Breast Cancer Growth by Facilitating CD8+ T Cell Infiltration and Inducing a Novel Set of Genes including Potential Tumor Suppressor Genes in 1q21.3
Source: Cancers (Basel). 2023 Jun 15;15(12):3203. doi: 10.3390/cancers15123203 (PMC10296577; doi:10.3390/cancers15123203)
Supplement: Supplementary file 1 [file cancers-15-03203-s001.zip › Sup Fig S1 rev.pdf]

Figure S1

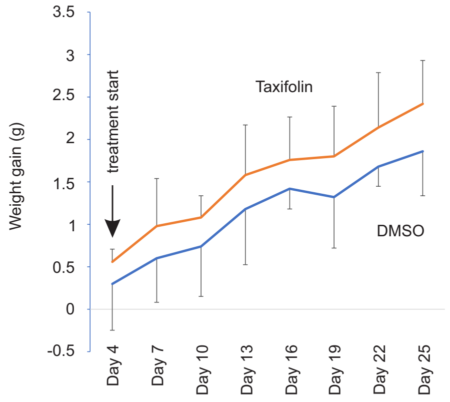

Figure S1. Taxifolin treatment did not result in weight loss. Tumor-free mice of 8-weeks old were administrated with DMSO (n = 5) or taxifolin (50 mg/kg, n = 5) via ip at the indicated time points. Animal weights were recorded. The mean animal weights at day 0 were 20.6 ± 2.5 (means ± SD) for the DMSO group and 20.9 ± 1.1 for the taxifolin group ($p = 0.84$). As the treatment started at day 4, the weight gains from day 0 to day 4 for both groups ($p = 0.39$) occurred naturally or without treatment; all weight gains were in refence to the base-line weights at day 0. The differences for weight gains between the 2 groups at individual time points were at $p > 0.16$ with the smallest p value being $p = 0.165$ for both groups at day 25.
