# Supplementary material for: Taxifolin Inhibits Breast Cancer Growth by Facilitating CD8+ T Cell Infiltration and Inducing a Novel Set of Genes including Potential Tumor Suppressor Genes in 1q21.3
Source: Cancers (Basel). 2023 Jun 15;15(12):3203. doi: 10.3390/cancers15123203 (PMC10296577; doi:10.3390/cancers15123203)
Supplement: Supplementary file 1 [file cancers-15-03203-s001.zip › Sup Fig S10 rev.pdf]

Figure S10

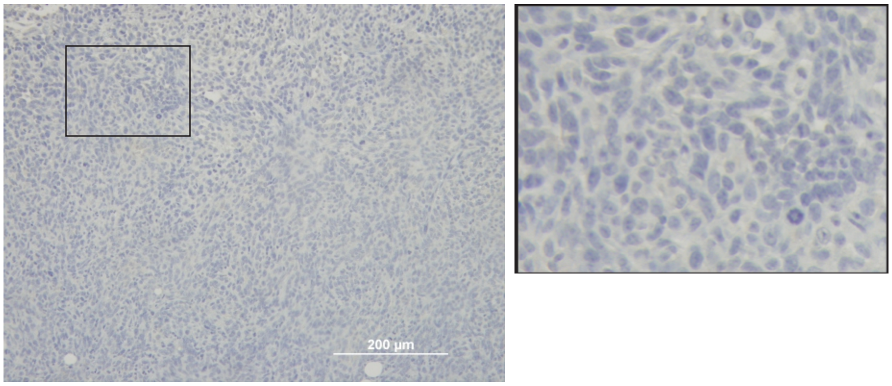

Figure S10. Typical IHC staining of 4T-1 tumors with control IgG. The marked region was enlarged 3 folds.
