# Supplementary material for: Taxifolin Inhibits Breast Cancer Growth by Facilitating CD8+ T Cell Infiltration and Inducing a Novel Set of Genes including Potential Tumor Suppressor Genes in 1q21.3
Source: Cancers (Basel). 2023 Jun 15;15(12):3203. doi: 10.3390/cancers15123203 (PMC10296577; doi:10.3390/cancers15123203)
Supplement: Supplementary file 1 [file cancers-15-03203-s001.zip › Sup Fig S3 rev.pdf]

Figure S3

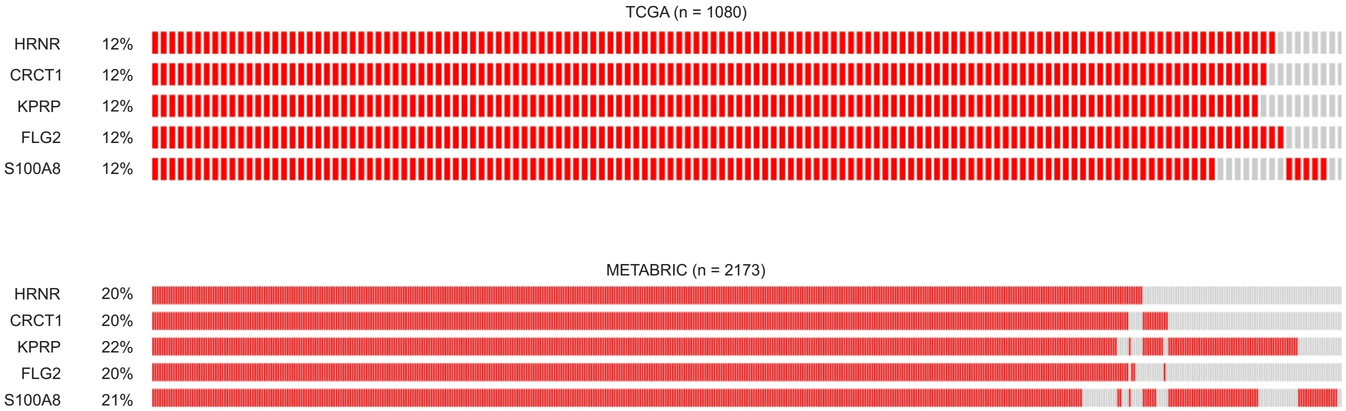

Figure S3. Copy number increases of the indicated genes in both TCGA and METABRIC datasets. The analyses were determined using tools provided by cBioPortal. Only the proportions of tumors with the indicated amplifications (red) are included.
