# Supplementary material for: Taxifolin Inhibits Breast Cancer Growth by Facilitating CD8+ T Cell Infiltration and Inducing a Novel Set of Genes including Potential Tumor Suppressor Genes in 1q21.3
Source: Cancers (Basel). 2023 Jun 15;15(12):3203. doi: 10.3390/cancers15123203 (PMC10296577; doi:10.3390/cancers15123203)
Supplement: Supplementary file 1 [file cancers-15-03203-s001.zip › Sup Fig S6_proof.pdf]

Figure S6

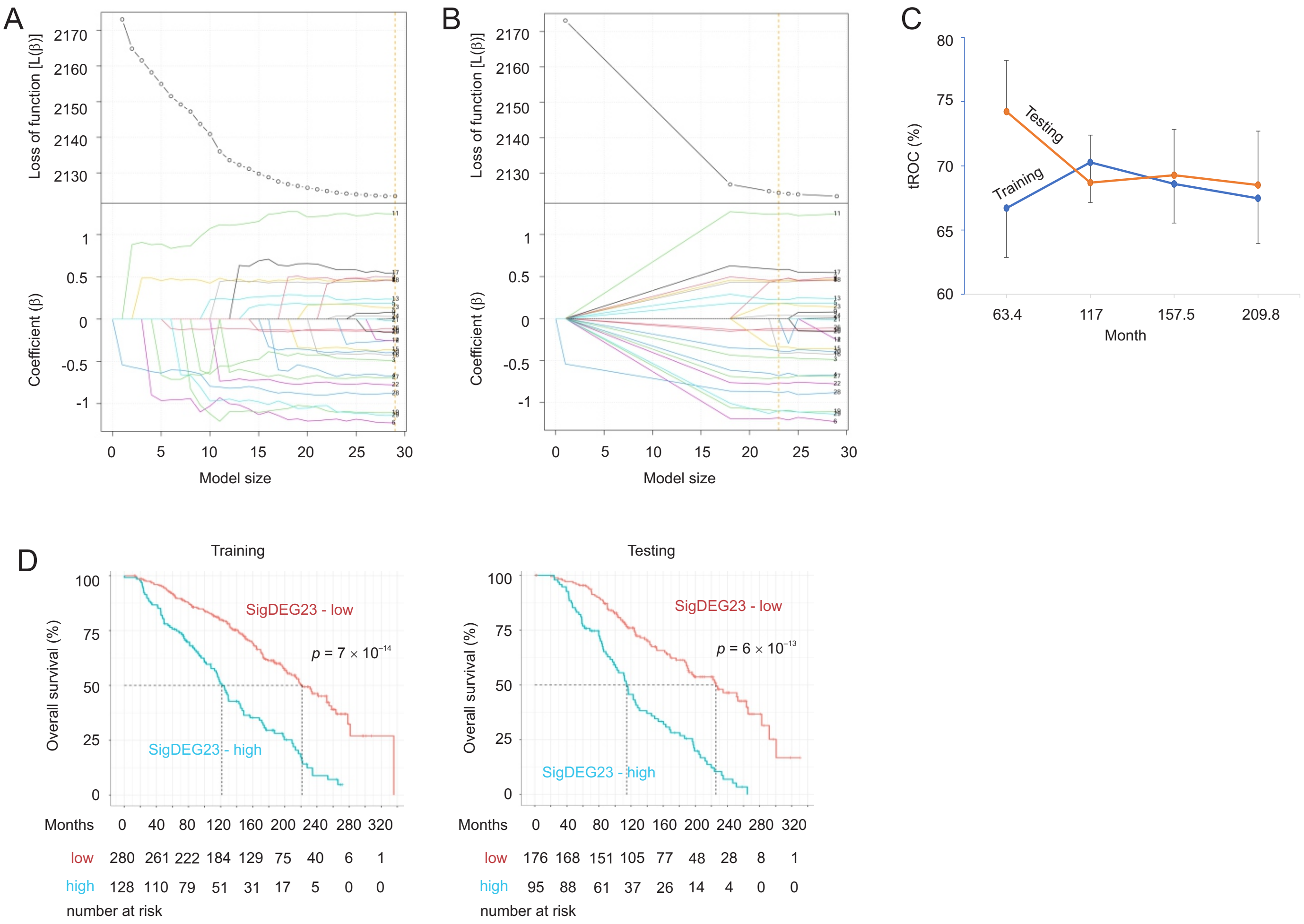

Months	0	40	80	120	160	200	240	280	320
low	280	261	222	184	129	75	40	6	1
high	128	110	79	51	31	17	5	0	0

Months	0	40	80	120	160	200	240	280	320
low	176	168	151	105	77	48	28	8	1
high	95	88	61	37	26	14	4	0	0

Figure S6. Construction and characterization of SigDEG23. (**A, B**) Optimization of DEG36 model size by Cox regression (BeSS R package) using sequential (A) and golden selection (gselection) (B) methods. The dotted line in panel B indicates the optimized model size (n = 23), which we named it as SigDEG23. (**C**) Time-dependent ROC-AUC curves for SigDEG23 in discrimination of the mortality risk in the Training and Testing sub-populations (see "Results" for details). (**D**) Stratification of BC mortality in Training and Testing populations. Cutoff points were estimated using Maximally Selected Rank Statistics. Statistical analysis was performed using log-rank test.
