# Supplementary material for: Taxifolin Inhibits Breast Cancer Growth by Facilitating CD8+ T Cell Infiltration and Inducing a Novel Set of Genes including Potential Tumor Suppressor Genes in 1q21.3
Source: Cancers (Basel). 2023 Jun 15;15(12):3203. doi: 10.3390/cancers15123203 (PMC10296577; doi:10.3390/cancers15123203)
Supplement: Supplementary file 1 [file cancers-15-03203-s001.zip › Sup Fig S7_proof.pdf]

Figure S7

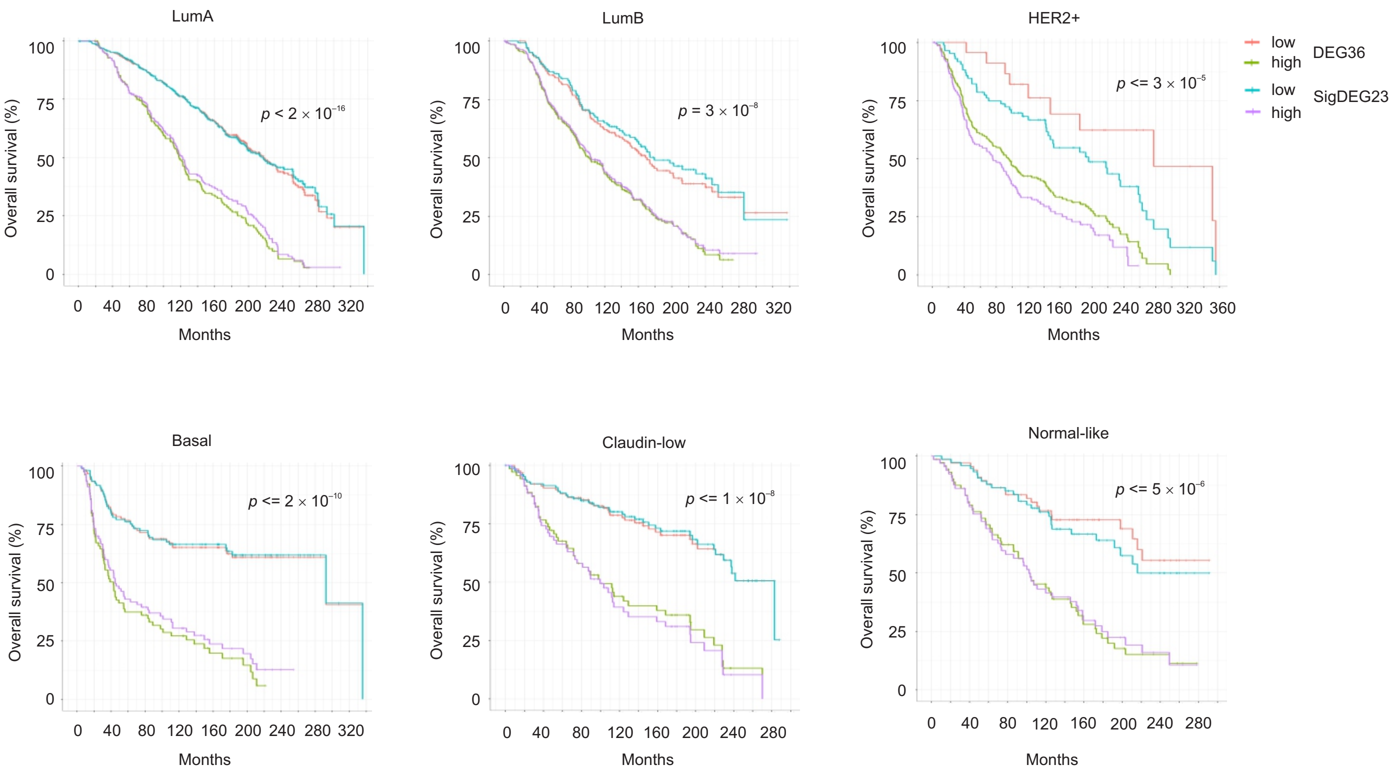

Figure S7. Stratification of poor OS by DEG36 and SigDEG23. Mortality risk separation by DEG36 and SigDEG23 in the indicated intrinsic BCs within the METABRIC dataset was performed with the R survival package using cutoff points estimated by Maximally Selected Rank Statistics. LumA: Luminal A; LumB: Luminal B; HER2+: HER2-enriched; Basal: Basal-like.
