# Supplementary material for: Taxifolin Inhibits Breast Cancer Growth by Facilitating CD8+ T Cell Infiltration and Inducing a Novel Set of Genes including Potential Tumor Suppressor Genes in 1q21.3
Source: Cancers (Basel). 2023 Jun 15;15(12):3203. doi: 10.3390/cancers15123203 (PMC10296577; doi:10.3390/cancers15123203)
Supplement: Supplementary file 1 [file cancers-15-03203-s001.zip › Sup Fig S8_proof.pdf]

Figure S8

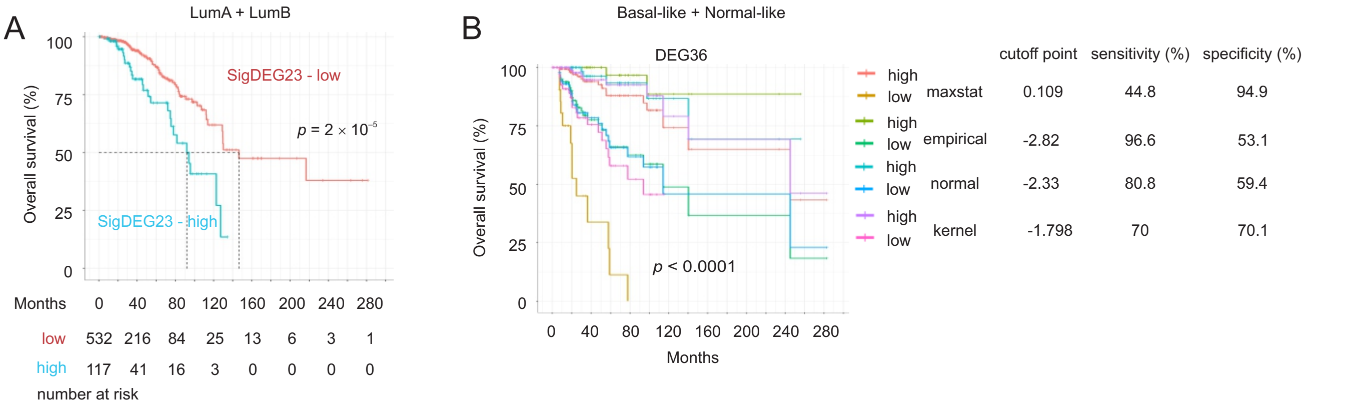

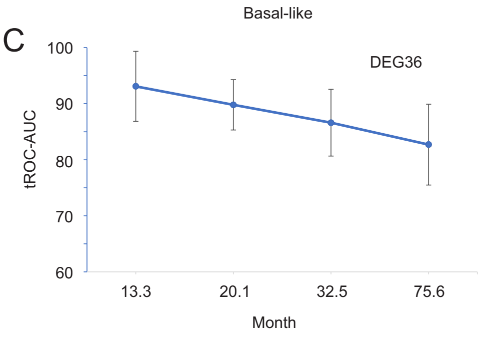

Figure S8. Characterization of SigDEG23 and DEG36. (**A**) Stratification of BC mortality risk in the LumA + LumB population within the TCGA dataset. (**B**) Cutoff points of DEG36 risk score in stratification of BC mortality in the Basal-like + Normal-like population within the TCGA dataset were estimated with the indicated methods. The corresponding survival curves were constructed with the relevant sensitivity and specificity included. (**C**) Time-dependent ROC-AUC for DEG36 in discrimination of poor OS in Basal-like BCs within the TCGA dataset.
