# Supplementary material for: Taxifolin Inhibits Breast Cancer Growth by Facilitating CD8+ T Cell Infiltration and Inducing a Novel Set of Genes including Potential Tumor Suppressor Genes in 1q21.3
Source: Cancers (Basel). 2023 Jun 15;15(12):3203. doi: 10.3390/cancers15123203 (PMC10296577; doi:10.3390/cancers15123203)
Supplement: Supplementary file 1 [file cancers-15-03203-s001.zip › Sup Fig S9.pdf]

Figure S9

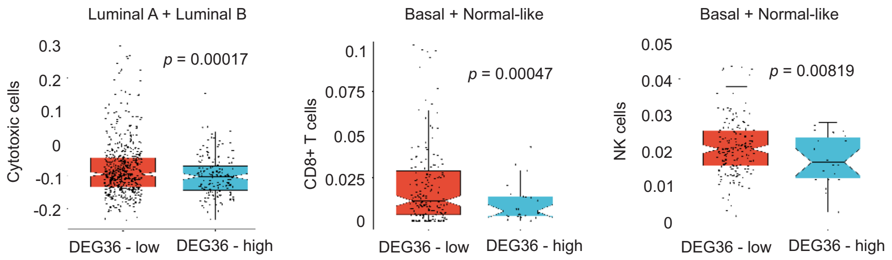

Figure S9. Association of DEG36 with the exclusion of cytotoxic lymphocytes from BC. The indicated immune cell contents in the indicated BCs within the TCGA dataset were determined using ssGSEA (the left panel) and quantiseq (the middle and right panels).
