# Supplementary material for: Taxifolin Inhibits Breast Cancer Growth by Facilitating CD8+ T Cell Infiltration and Inducing a Novel Set of Genes including Potential Tumor Suppressor Genes in 1q21.3
Source: Cancers (Basel). 2023 Jun 15;15(12):3203. doi: 10.3390/cancers15123203 (PMC10296577; doi:10.3390/cancers15123203)
Supplement: Supplementary file 1 [file cancers-15-03203-s001.zip › Table S1.pdf]

Table S1. Real-time PCR primers used to amplify the indicated DEGs

Gene	Forward primer	Reverse primer
Cd209e	AGATGGGCTCTCTGGGATTT	GCAGGCAGTCATGGAGTCAT
Cyp2f2	TTCCTGATACCCAAGGGCAC	CTGAGGCGTCTTGAACTGGT
Atp6v1c2	CTTCCGTCGCTCCTGTCTTG	GCTGCTTGGCGGGATATTTG
Sbsn	GGACAAAGTAGCCCACGACA	CCTTGATGAGACCCCTGTCC
Ly6g6c	ACAAACGTGTACCTTGGGAAG	TGCAATAACCAGAGGCCGAG
Lce1m	CTCCAGTGCCTGAGTTCCTT	GGCACTTTGGGGTCTTGCAT
Kprp	TGCTGAGGAGGGTCTTGCT	CGGCACACAACACTGAATCG
Sprr2a3	CTCCGGAGAACCTGATTCTGA	GCTATGGAGTCGGTGAGGTG
Hrnr	TGCAAGCAACATCAGTCTCCA	GGTGTCTGGATCATCTGGATTCTTC
Serpinb3b	GCCACCATGATTCGTTTTCATGC	GGACATTTTCTTCATCATCGCAGT
