# Supplementary figures and images for: Taxifolin Inhibits Breast Cancer Growth by Facilitating CD8+ T Cell Infiltration and Inducing a Novel Set of Genes including Potential Tumor Suppressor Genes in 1q21.3
Source: Cancers (Basel). 2023 Jun 15;15(12):3203. doi: 10.3390/cancers15123203 (PMC10296577; doi:10.3390/cancers15123203)

Figure S4

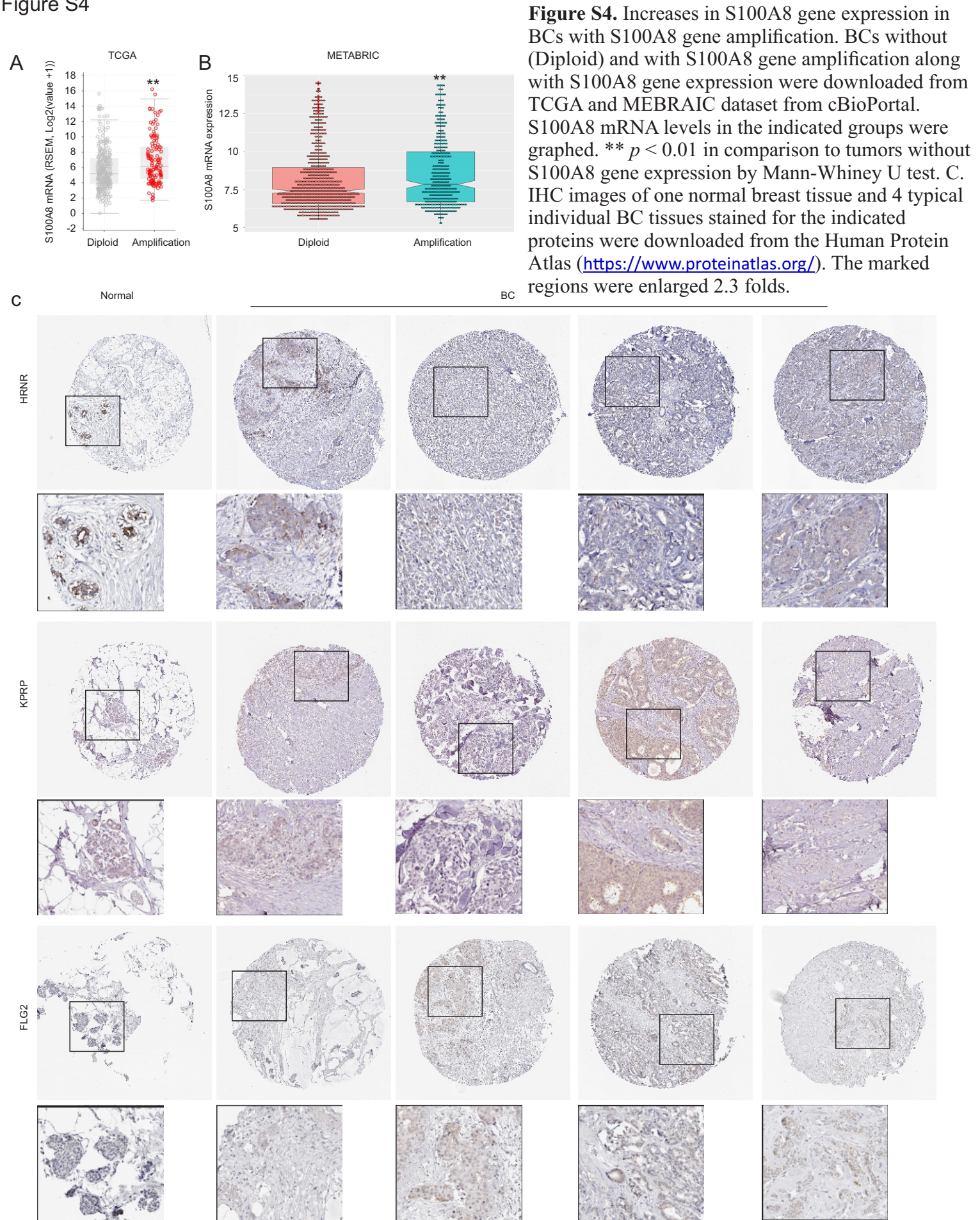

Supplement: Supplementary file 1 [file cancers-15-03203-s001.zip › Sup Fig S4.pdf]
